# Psychosis and Incidental Latent Toxoplasmosis: A Case Report and Key Learning Points

**DOI:** 10.7759/cureus.104920

**Published:** 2026-03-09

**Authors:** Filipa M.A.A. Teixeira

**Affiliations:** 1 Psychiatry, South West London and St. George's Mental Health NHS Trust, London, GBR; 2 Addiction Clinical Care Suite, Guy's and St Thomas' NHS Foundation Trust, London, GBR; 3 Clinical Trials Facility, King's College London, London, GBR

**Keywords:** clinical decision-making, diagnostic reasoning, incidental findings, latent toxoplasmosis, long-acting injectable antipsychotics, organic psychosis differential, psychotic relapse, schizophrenia spectrum, serological interpretation, substance-induced exacerbation

## Abstract

Psychotic disorders are characterized by hallucinations, delusions, and impairments in reality testing, with relapses often associated with medication non-compliance, psychiatric stressors, and substance use. *Toxoplasma gondii* is a neurotropic intracellular parasite with a high global seroprevalence, and latent infection has been documented in association with psychotic syndromes, although the nature of this relationship remains uncertain. The aim of this case report is to describe a patient presenting with a psychotic relapse in whom latent *Toxoplasma gondii* infection was identified during inpatient investigation.

This is a case of a 37-year-old woman who presented to the emergency department with a severe psychotic relapse on a background characterized by persecutory delusions, sexual misinterpretations, multimodal hallucinations (including visual phenomena), and substantial functional deterioration. Her psychiatric history included a background of unspecified primary psychosis with multiple admissions due to psychotic relapses, substance misuse (alcohol and amphetamines), and periods of disengagement with mental health services. During admission, a palpable cervical nodule was identified, prompting further investigations, which revealed previous exposure to *Toxoplasma gondii.* Routine physical examination revealed a palpable cervical nodule, and given the context of cat exposure, serological examination was ordered. This revealed a latent *Toxoplasma gondii* infection (IgG positive, IgM negative). She did not have any neurological features consistent with toxoplasmic encephalitis and did not require antiparasitic therapy. Recent use of stimulants and alcohol use before admission were acknowledged. She was started on oral risperidone and then transitioned to paliperidone palmitate on account of symptom severity and concerns about adherence. She significantly improved, with complete resolution of psychotic symptoms. She was discharged on depot treatment, with continued community support. This case illustrates caution in interpreting incidental findings in complex psychotic manifestations.

Even though associations between latent *Toxoplasma gondii* and psychosis have been documented, the nature of this relationship remains uncertain. This case highlights the need to interpret a past *Toxoplasma gondii *infection with caution and the importance of considering contributing factors such as substance misuse and psychosocial stressors.

## Introduction

Psychotic disorders represent a big proportion of mental health comorbidity and are characterised by hallucinations, delusions, and impairments in reality testing [1]. Hallucinations correspond to experiences related to changes in perception, without a stimulus, which can be visual, auditory, tactile, gustatory, or olfactory in nature. In psychotic disorders, auditory hallucinations are more common, and the presence of visual hallucinations should prompt exclusion of neurological conditions (e.g., encephalitis, brain tumours, epilepsy, migraines, seizures, Parkinson's disease, Lewy body dementia, narcolepsy), central nervous system infections, retinal pathology, endocrine disturbances, alcohol withdrawal, and autoimmune disorders. In these cases, further investigations may be needed, such as imaging, specialist input, neurological examination or further laboratory investigations. Red flags would include prominent visual hallucinations, fluctuating level of consciousness, acute onset, neurological deficits, cognitive impairment, abnormal vital signs, or associated systemic symptoms. Delusions are fixed, false beliefs strongly held against evidence and not in keeping with cultural and religious norms. Both are core features of psychotic disorders as described in disease classification systems.

According to the Diagnostic and Statistical Manual of Mental Disorders, Fifth Edition (DSM-5) [2], psychotic disorders are defined by the presence of core symptoms such as delusions, hallucinations, disorganised thinking (speech), grossly disorganised or catatonic behaviour, and negative symptoms, and the International Classification of Diseases, 11th Revision (ICD-11) describes psychotic disorders, including "Schizophrenia or other primary psychotic disorder, unspecified" (6A2Z), as conditions characterised by impaired thinking and altered perception, alongside delusions and impairment in functioning [3]. Relapses are not uncommon and are usually associated with drug non-compliance, psychiatric stressors, and comorbid substance use. Stimulant and alcohol use in particular are well-established factors that can worsen severity, impair insight, and increase the likelihood of admissions and worse clinical outcomes [4]. 

In inpatient admissions, investigations and examinations are common practice to exclude organic causes of psychiatric presentations, including, for example, neurological, infectious, or metabolic conditions.

*Toxoplasma gondii *is a neurotropic intracellular parasite with a global adult seroprevalence estimated between 30% and 50% [5]. Acute toxoplasmosis occurs predominantly in immunocompromised patients with focal neurological symptoms and behavioural disturbances, characterised by toxoplasmic encephalitis [6]. In contrast, latent toxoplasmosis is characterised by isolated IgG seropositivity in immunocompetent individuals and has been widely documented in association with psychotic syndromes.

In case-control studies, *Toxoplasma gondii* IgG seropositivity has been found to be higher in populations affected by schizophrenia compared to healthy controls [7]. These findings have been supported by large systematic reviews and meta-analyses showing a relatively small but reproducible association between latent toxoplasmosis and psychotic disorders [7, 8].

Most recently, data from longitudinal cohorts have shown a higher incidence of schizophrenia in seropositive individuals [9]. Hypothesised mechanisms range from immune-mediated neuroinflammation to parasite-related changes in dopaminergic signalling, although exact mechanisms are still unclear [10].

Even though associations between *Toxoplasma gondii *infection and psychosis have been documented, the nature of this relationship is uncertain; therefore, serological findings should be interpreted with caution in patients with psychosis, especially if within the context of substance use. 

Furthermore, the clinical significance of positive serology in patients presenting with psychotic symptoms remains unclear in routine psychiatric practice.

This case report describes a patient presenting with a severe psychotic relapse and prominent visual hallucinations in whom *Toxoplasma gondii *seropositivity, indicating previous infection, was identified during the diagnostic work-up, highlighting the challenges of interpreting findings in the context of multiple psychiatric and substance-related risk factors.

## Case presentation

We report the case of a 37-year-old woman who presented to the emergency department due to a psychotic relapse with impaired functioning.

In hospital, she presented as extremely distressed and guarded, and her thoughts were dominated by beliefs that she was being monitored, sexually targeted (persecutory delusions), and inherently relevant to powerful people or organisations (grandiose delusions). For example, she believed that events around her were connected to powerful groups and stated that “important people are involved in this” and that she had become a central figure in a wider conspiracy. She reported that others were monitoring her because she was “important to them” and that her actions had wider consequences beyond her immediate environment. Her persecutory beliefs included statements such as “somebody was trying to kill me” and that there was “something very evil in my flat.” She believed that there was “a cult in my building” and that a woman had been killed and “her body is hidden in my walls.” She described the walls as feeling “soft like sand,” which she interpreted as evidence of this belief. She also reported that people were able to monitor her thoughts and actions, stating that others could “hear my thoughts” and that someone had implanted something in her face that allowed them to control or monitor her. She also described experiences consistent with passivity phenomena, reporting that external forces were influencing her thoughts and behaviour. For example, she stated that voices were “controlling me” and making her perform actions such as digging into the walls. On other occasions, she reported that she was “controlled” and acting under external influence.

She had commanding hallucinations, including voices directly telling her to “dig a hole in the wall” and to “set a fire to blow up the building". She did not hear voices speaking in the third person. Staff also observed behaviour consistent with responding to unseen stimuli. The patient described visual phenomena associated with her beliefs, including seeing “evil eyes” in her room and visions of people “cutting off my head and burning my body.” She also reported tactile sensations without an external stimulus, describing experiences where she felt “a big force penetrating me” despite nobody being present. These experiences were interpreted by the patient as evidence that she had been sexually assaulted by staff. These visual hallucinations were consistent with those previously exhibited in previous psychotic episodes. She presented with disorganised behaviour, impaired self-care, disturbed sleep, and reduced food and fluid intake. Her insight was significantly impaired. 

Her background history included significant early-life trauma. Records indicated repeated sexual abuse during childhood and adolescence, as well as later experiences of domestic violence in adult relationships. These experiences contributed to longstanding emotional dysregulation and vulnerability to mental health difficulties.

The patient had a long-standing history of substance use, primarily involving amphetamines and alcohol. She reported first using amphetamines during adolescence (approximately age 14) and had periods of daily use in the past, typically administered intranasally. During some periods, she was using amphetamines up to twice daily. In the months preceding admission, her use had become intermittent; however, in the weeks prior to admission, stimulant use had escalated again to approximately weekly use, although she could not quantify her use. This was due to psychosocial stressors, including social isolation and relationship difficulties. She reported that amphetamines helped her feel more focused and organised her thoughts, although clinicians considered that stimulant use likely exacerbated paranoia and behavioural disorganisation. Her pattern of use was hazardous, as it placed her at risk of harm despite no clear evidence of established dependence. Alcohol use was also reported, typically involving up to seven 750-ml beers per week with occasional heavier intake. She did not meet the criteria for alcohol dependence, and her use was not harmful but rather hazardous in nature. Psychotic symptoms had been documented both during periods of stimulant use and during periods when stimulant use was not clearly temporally related to symptom onset. She also smoked approximately six to 20 cigarettes daily.

She lived alone with a domestic cat and reported prolonged exposure to cats over several years. Regarding her medical history, she had a background of asthma and allergic rhinitis with no history of immunocompromising conditions, malignancy, or human immunodeficiency virus infection. Her regular medications when admitted were inhaled beclometasone and salbutamol for asthma, taken when needed, and quetiapine, as above. She had no known drug allergies. She had no family history of psychosis or of other mental health issues, to her knowledge.

Her psychiatric history was marked by multiple psychotic episodes over the previous years, as well as periods of disengagement from services. She was first admitted to a psychiatric hospital at the age of 13 following a suicide attempt. Over the past three years, she had multiple episodes characterised by auditory and visual hallucinations (involving figures or shapes emerging from walls or mirrors, including woman-like forms and “devils"), paranoid beliefs with supernatural themes, and fears of being watched or controlled. Symptoms fluctuated over time, seemingly due to emotional distress and associated with worsening drug use. Six to eight months preceding the admission, she presented with hallucinations and paranoia during community follow-up, but did not need to be admitted to the hospital. She had never been admitted to a mental health hospital between the ages of 13 and the current admission. The patient had previously been treated with aripiprazole (7.5 to 15 mg daily) for four years, up to two years before the current presentation. At this time, this was discontinued due to perceived loss of efficacy and replaced with quetiapine, titrated up to 100 mg daily.

She was detained for psychiatric assessment and treatment under mental health legislation, following a period of gradual deterioration over the preceding three months, as she progressively became more thought-disordered and paranoid, believing others were monitoring or controlling her, and frequently refused prescribed medication due to fears it had been poisoned. Her overall functioning and self-care had also declined significantly. On the day before admission, she self-presented to the police station, very distressed and paranoid, reporting that someone was performing voodoo on her and that others were able to read her thoughts. She described multiple bizarre and persecutory beliefs, including that neighbours were part of a cult and that supernatural forces were controlling her life. She was subsequently detained by police, brought to the emergency department, and then detained in an acute mental health hospital.

At the time of admission, she had been prescribed quetiapine at a dose of 100 mg at night by her community team, but did not take the medication consistently. This medication was switched to oral risperidone (1 mg once daily) and gradually increased to 4 mg daily over several days. Clinical response to treatment was subpar, and compliance with medication was queried.

At one week of admission, in the context of a routine physical examination, a palpable, mobile, non-tender, and painless nodule was identified, measuring approximately 1 cm in diameter. Given this finding, and given a history of being in close contact with multiple cats constantly since childhood and at present, further investigations were conducted to exclude organic causes for her presentation, especially given the unusual presentation of visual hallucinations and poor response to treatment. In primary psychotic disorders such as schizophrenia, hallucinations are most commonly auditory, whereas prominent visual hallucinations may raise suspicion for secondary or organic causes of psychosis, including neurological, infectious, or inflammatory conditions. Infection by *Toxoplasma gondii *can cause inflammation in the visual association cortex (occipito-temporal regions) or in thalamocortical circuits, which may result in visual hallucinations. In this case, the patient described vivid visual phenomena, including seeing “evil eyes” in her room and visions of people “cutting off my head and burning my body.” Given the patient’s history of extensive cat exposure and the presence of a characteristic nodule, investigations were performed to exclude infectious causes such as *Toxoplasma gondii *infection.

Serological examination showed previous infection with *Toxoplasma gondii*, cytomegalovirus, and Epstein-Barr virus. Furthermore, she did not show any focal neurological signs, nor did she display symptoms suggestive of toxoplasmic encephalitis.

Differential diagnoses included drug-induced psychosis in the context of stimulant use, affective psychosis such as bipolar disorder with psychotic features given grandiosity and disinhibition, trauma-related phenomena given her extensive abuse history, and potential organic causes of psychosis. However, the persistence of fixed delusions, multimodal hallucinations, and passivity experiences beyond periods of intoxication or withdrawal, together with progressive deterioration in functioning, seemed more consistent with a primary psychotic disorder.

Given the poor response to treatment and concerns around adherence, as she continued experiencing persecutory delusions, command auditory hallucinations, and behavioural disorganisation, her oral risperidone was switched to paliperidone palmitate long-acting intramuscular injection at doses of 150 mg in the loading regimen, followed by 100 mg one week later. Her symptoms began to gradually improve, as there was a robust reduction in the intensity of her persecutory and grandiose beliefs, her hallucinations resolved, and her insight improved. Although her mental state had stabilised, she developed dose-related side effects: daytime sedation, subjective emotional blunting, and mild extrapyramidal symptoms, including subjective rigidity and inner restlessness. As a result, her maintenance dose was adjusted to 75 mg monthly, and she was discharged on this dose. She remained stable on paliperidone palmitate injections in the community, with monthly follow-up for depot administration and relapse prevention, as well as support with dealing with anxiety. Table 1 illustrates the timeline of the patient's psychiatric history, including key events from the current admission through to discharge.

**Table 1:** Timeline on psychiatric history and key events during admission

Time	Event
Adolescence	Onset of amphetamine use
Age 13	Suicide attempt and first psychiatric admission
2017	First known to mental health services
January-February 2025	Hallucinations and paranoia were noted in the community
May 2025	Increasing disorganisation and persecutory delusions
August 2025	Presents to the police
August 30, 2025	Admitted to an acute mental health hospital
Week 1 admission	Neck lymph node identified
Week 1 admission	*Toxoplasma *IgG positive
Week 2 admission	Paliperidone depot initiated
Discharge (after 16 days from admission)	Symptoms resolved with depot treatment

Investigations

On admission, baseline laboratory investigations were conducted. Full blood count was within reference range. Renal function and electrolytes were normal with creatinine 73 µmol/L (reference range 44-80 µmol/L) and estimated glomerular filtration rate >90 mL/min/1.73 m². The liver function test and thyroid testing were normal. Random plasma glucose was mildly elevated at 8.3 mmol/L (reference range 3.5-5.4 mmol/L), and glycated haemoglobin was 34 mmol/mol (reference range 20-41 mmol/mol), consistent with stress-induced hyperglycaemia rather than diabetes mellitus. Creatine kinase was at the upper limit of normal at 197 U/L (reference range 25-200 U/L). C-reactive protein was not increased at the level of 1.6 mg/L (reference range 0-5 mg/L). Iron testing revealed low serum iron at 10 µmol/L (reference 14-30 µmol/L) and normal transferrin saturation at 16% (reference range 15%-50%) and ferritin over the reference range at 101 µg/L (reference range 15-98 µg/L), without evidence of iron deficiency or active inflammatory disease. Vitamin D was low at 31 nmol/L (reference range 50-174 nmol/L), which is indicative of vitamin D insufficiency. Coagulation investigations performed following admission were within normal limits, due to epistaxis, but these were normal. Laboratory investigation results are summarised in Table 2.

**Table 2:** Blood investigations at admission Abbreviations: MCV, mean corpuscular volume; MCH, mean corpuscular haemoglobin; MCHC, mean corpuscular haemoglobin concentration; RDW, red cell distribution width; MPV, mean platelet volume; eGFR (EPI), estimated glomerular filtration rate calculated using the Chronic Kidney Disease Epidemiology Collaboration (CKD-EPI) equation; ALP, alkaline phosphatase; ALT, alanine aminotransferase; HDL, high-density lipoprotein; LDL, low-density lipoprotein; TSH, thyroid-stimulating hormone; HbA1c (IFCC), glycated haemoglobin reported in International Federation of Clinical Chemistry units.

Category	Parameter	Result	Units	Reference range
Full blood count	White cell count	10.8	×10⁹/L	4.0–11.0
	Haemoglobin	138	g/L	115–165
	Platelets	364	×10⁹/L	150–450
	Red blood cells	4.66	×10¹²/L	3.8–5.8
	Haematocrit	0.397	L/L	0.37–0.47
	MCV	85.2	fL	78–97
	MCH	29.6	pg	27–34
	MCHC	347	g/L	310–360
	RDW	13.5	%	11.5–15
	MPV	7.6	fL	Not established
	Neutrophils	7	×10⁹/L	1.5–8.0
	Lymphocytes	2.7	×10⁹/L	1.1–4.0
	Monocytes	0.9	×10⁹/L	0.2–1.1
	Eosinophils	0.2	×10⁹/L	0.1–0.4
	Basophils	0	×10⁹/L	0–0.3
Metabolic/renal	Plasma glucose	8.3	mmol/L	3.5–5.4
	Sodium	138	mmol/L	133–146
	Potassium	3.8	mmol/L	3.5–5.3
	Urea	5.7	mmol/L	2.5–7.8
	Creatinine	73	µmol/L	44–80
	eGFR (EPI)	>90	mL/min/1.73m²	>90
Liver function	Albumin	41	g/L	35–50
	Bilirubin	9	µmol/L	0–21
	ALP	79	U/L	30–130
	ALT	14	U/L	0–40
Inflammation/muscle	C-reactive protein	1.6	mg/L	0–5
	Creatine kinase	197	U/L	25–200
Lipid profile	Total cholesterol	3.8	mmol/L	3.3–5.2
	HDL cholesterol	1.36	mmol/L	1.1–2.6
	LDL cholesterol	1.91	mmol/L	<3.0
	Non-HDL cholesterol	2.44	mmol/L	Not established
	Triglycerides	1.29	mmol/L	<1.7
	Cholesterol: HDL ratio	2.8	ratio	0–5
Bone/endocrine	Calcium	2.41	mmol/L	2.2–2.6
	Adjusted calcium	2.39	mmol/L	2.2–2.6
	Phosphate	1.23	mmol/L	0.8–1.5
	TSH	1.86	mIU/L	0.27–4.2
	HbA1c (IFCC)	34	mmol/mol	20–41
Iron studies	Serum iron	10	µmol/L	14–30
	Transferrin	2.46	g/L	2.0–3.6
	Transferrin saturation	16	%	15–50
	Ferritin	101	µg/L	15–98
	Total iron-binding capacity	62	µmol/L	50–85
Total 25-hydroxyvitamin D	Total 25-hydroxyvitamin D	31	nmol/L	50 - 174

About a week after admission, a small, mobile, non-tender nodule was found in the neck region, which led to further focused investigations being performed. Serological testing showed positive IgG and negative IgM antibodies against *Toxoplasma gondii*, compatible with prior exposure. Cytomegalovirus and Epstein-Barr virus serology also showed positive IgG, positivity, and negative IgM, implying prior infection and no acute viral disease. Laboratory investigation results are summarised in Table 3.

**Table 3:** Blood investigations on day 8 Abbreviations: RBC, red blood cells; MCV, mean corpuscular volume; MCH, mean corpuscular haemoglobin; MCHC, mean corpuscular haemoglobin concentration; RDW, red cell distribution width; MPV, mean platelet volume; INR, international normalised ratio; APTT, activated partial thromboplastin time; APTT ratio, activated partial thromboplastin time ratio; eGFR (EPI), estimated glomerular filtration rate calculated using the Chronic Kidney Disease Epidemiology Collaboration (CKD-EPI) equation; ALP, alkaline phosphatase; ALT, alanine aminotransferase; HDL, high-density lipoprotein; LDL, low-density lipoprotein; TSH, thyroid-stimulating hormone; IgM, immunoglobulin M; IgG, immunoglobulin G; EBV, Epstein–Barr virus; VCA, viral capsid antigen; EBNA, Epstein–Barr nuclear antigen.

Category	Parameter	Result	Units	Reference range
Full blood count	White cell count	8.6	×10⁹/L	4.0 – 11.0
	Haemoglobin	134	g/L	115 – 165
	Platelets	312	×10⁹/L	150 – 450
	RBC	4.48	×10¹²/L	3.8 – 5.8
	Haematocrit	0.393	L/L	0.37 – 0.47
	MCV	87.7	fL	78 – 97
	MCH	29.9	pg	27 – 34
	MCHC	341	g/L	310 – 360
	RDW	13.1	%	11.5 – 15.0
	MPV	7.4	fL	Not stated
	Neutrophils	6	×10⁹/L	1.5 – 8.0
	Lymphocytes	1.9	×10⁹/L	1.1 – 4.0
	Monocytes	0.6	×10⁹/L	0.2 – 1.1
	Eosinophils	0.1	×10⁹/L	0.1 – 0.4
	Basophils	0	×10⁹/L	0.0 – 0.3
Coagulation screen	Prothrombin time	9.8	s	9 – 13
	INR	0.87	ratio	0.8 – 1.1
	APTT	30.8	s	26 – 36
	APTT ratio	1.07	ratio	0.85 – 1.15
Metabolic/renal	Sodium	139	mmol/L	133 – 146
	Potassium	3.9	mmol/L	3.5 – 5.3
	Urea	5	mmol/L	2.5 – 7.8
	Creatinine	73	µmol/L	44 – 80
	eGFR (EPI)	>90	mL/min/1.73 m²	>90
Liver function	Albumin	36	g/L	35 – 50
	Bilirubin	4	µmol/L	0 – 21
	ALP	71	U/L	30 – 130
	ALT	15	U/L	0 – 40
Lipid profile	Total cholesterol	4.6	mmol/L	3.3 – 5.2
	HDL cholesterol	1.22	mmol/L	1.1 – 2.6
	Non-HDL cholesterol: HDL ratio	3.38 3.8	mmol/L —	Not stated 0 – 5
	Triglycerides	2.19	mmol/L	<1.7*
	LDL cholesterol	2.52	mmol/L	Not stated
Bone profile	Calcium	2.18	mmol/L	2.2 – 2.6
	Adjusted calcium	2.26	mmol/L	2.2 – 2.6
	Phosphate	1.25	mmol/L	0.8 – 1.5
Thyroid function tests	TSH	1.18	mIU/L	0.27 – 4.2
Infectious serology	Toxoplasma IgM	Not detected	—	—
	Toxoplasma IgG	Detected	—	—
	Cytomegalovirus IgM	Not detected	—	—
	Cytomegalovirus IgG	Detected	—	—
	EBV VCA IgM	Not detected	—	—
	EBV VCA IgG	Detected	—	—
	EBV EBNA IgG	Detected	—	—

We intended to exclude structural or inflammatory pathology on magnetic resonance imaging of the brain; however, clinical improvement, distress about further investigations, and the lack of a clear additional diagnostic benefit meant that imaging was not pursued. 

Differential diagnoses

The primary diagnosis was schizophrenia or another primary psychotic disorder, unspecified (ICD-11: 6A2Z) [3]. This was marked by persistent persecutory and delusions of reference (including the continuous monitoring through mirrors, phones and household fixtures), multimodal hallucinations (auditory, visual and tactile), passivity phenomena and behavioural disorganisation. These symptoms occurred beyond the time of acute intoxication, were associated with impaired insight, and responded well to antipsychotic treatment, all suggestive of a primary psychotic illness.

A presentation of substance-induced psychosis was carefully considered in light of the history of stimulant use. Amphetamine use triggers paranoia, hallucinations, and agitation and was probably a factor in exacerbating symptoms. However, psychotic episodes had been present before the recent escalation in drug use and were not previously temporally associated with alcohol or substance use. Symptoms also persisted beyond the expected period of intoxication or withdrawal and improved in a sustained manner with antipsychotic therapy, rather than simply cessation of substances. This significantly reduced the likelihood of substance use alone explaining her presentation.

Affective psychosis, such as bipolar disorder with psychotic features or schizoaffective disorder, was also considered, given historical accounts of episodes of mood fluctuations, grandiosity, and impulsive spending. At the time of admission, however, psychotic symptoms did not arise following an obvious mood episode, and there was no clearly documented history of mood episodes sustained in time, but rather fluctuations within the same day.

The patient’s extensive trauma history and previous diagnosis of emotionally unstable personality disorder required careful differentiation from trauma-related intrusions or emotional dysregulation. Although anxiety, interpersonal difficulties, and self-harm risk suggested this, the existence of fixed bizarre delusions, passivity experiences, and multimodal hallucinations made this less likely to be a sole contributing factor for her presentation.

Finally, organic causes for psychosis were also considered. Infective and metabolic investigations did not indicate an acute organic process, and serology was suggestive of past infection rather than active infection, as above. Without focal neurological signs and with an obvious response to antipsychotic treatment, organic aetiology seemed unlikely.

Treatment

At admission, the patient presented with acute psychotic features, including significant persecutory delusions, hallucinations, behavioural disturbance, little insight, and a high level of risk. At first, management was geared towards quick symptomatic stabilisation, management of risks, and containment of risks in the ward. Pharmacological therapy began with switching from prescribed quetiapine 100 mg once daily (which she was not consistently taking) to oral risperidone, selected for its well-established effectiveness in first-line treatment of psychosis and good tolerability. Risperidone was commenced at 1 mg daily and titrated to 4 mg daily stepwise during the admission according to clinical response and tolerability. This led to some improvement of psychotic symptoms, but consideration had to be given to adherence, given her well-established non-concordance to the oral medication and past relapses associated with disengagement.

Considering this, and only when her mental status had been stabilised to allow for meaningful discussion, a decision was made that her long-acting injectable antipsychotic was to be switched over. She was started on paliperidone palmitate, and the loading dose was 150 mg intramuscularly, 100 mg intramuscularly the following week. After stabilisation on depot treatment, the maintenance dose was switched to 75 mg intramuscularly monthly, partly due to reported side effects such as sedation and subjective psychomotor slowing. An adjunctive treatment of 25 mg promethazine was used as needed for anxiety, agitation, and sleep disturbance in the acute phase. Thiamine 50 mg four times daily was started due to her history of alcohol dependence.

Key management strategies included non-pharmacological interventions. This involved structured nursing care, regular checking of the patient's mental state, psychoeducation targeting illness awareness and relapse prevention, and participation of the family to facilitate engagement and discharge planning. Social and vocational problems were addressed by multidisciplinary input, including recommendations for psychological counselling and community support for housing and financial support.

The patient was discharged home to the community mental health team for continuing follow-up for monthly paliperidone palmitate administration, continued physical health monitoring, and ongoing psychosocial support.

Outcome and follow-up

The patient was discharged after a 16-day-long inpatient admission, during which her acute psychotic symptoms stabilised with antipsychotic treatment and a transition to long-acting injectable therapy. She was clinically improved at the time of discharge, with complete resolution of hallucinations, significant reduction in persecutory beliefs, improved insight into her illness, and reduced risk to herself and other people. Paliperidone palmitate 75 mg intramuscularly monthly was recommended as a maintenance treatment, and she also was given thiamine 50 mg four times daily, asthma inhalers, oral iron supplementation and vitamin D replacement, given her history of poor food and fluid intake, uncertainty around the amount of alcohol intake (reported as ranging from approximately 750 ml of beer per week to heavier intake, though she struggled to quantify this), and results from blood investigations. 

At discharge, she was referred to a mental health team in the community to continue with monthly depot administration and physical health monitoring (weight, metabolic profile, assessment of prolactin-related symptoms, and an annual ECG) due to long-term antipsychotic use. Her general physician (GP) was advised to repeat iron studies, calcium and lipid profiles, and to continue routine physical health screening. For the following two-month follow-up, the patient maintained good engagement with community services and was concordant with depot treatment, with no recurrence of psychotic symptoms, although anxiety was still apparent but less impairing. She continued struggling with motivation, but with no impairment in activities of daily living. She said she was interested in getting back into low-stimulus work and was referred to vocational and employment support services. Psychological therapy has been arranged to work through residual anxiety and trauma-related symptoms.

## Discussion

This case serves to illustrate the challenge of interpreting latent *Toxoplasma gondii* infection in the context of an acute psychotic episode. Although the patient's symptoms were treated as a primary psychotic disorder, the incidental finding of *Toxoplasma gondii* IgG positivity prompted discussion as to its role in the patient’s presentation. *Toxoplasma gondii* is an intracellular protozoan that is neurotropic and establishes a lifelong latent infection after first exposure. In immunocompetent individuals, this has been mainly represented in the form of isolated IgG seropositivity independent of IgM, but does not typically result in overt neuropsychiatric illness if immunosuppression or focal neurological effects do not occur [5,6]. Classical toxoplasmic encephalitis, including seizures, focal deficits, altered consciousness, and radiological abnormalities, was absent in this case, which made an acute infective psychosis unlikely [6].

An association between *Toxoplasma gondii *infection and schizophrenia-spectrum disorders is well described in the literature. Large-scale systematic reviews and meta-analyses have shown higher *Toxoplasma gondii* IgG seropositivity in individuals suffering from schizophrenia compared to healthy controls, with a small but repeatable effect in the population [7]. Recent studies have pointed to *Toxoplasma gondii *as a risk factor for schizophrenia among different environmental and infectious exposures and emphasised heterogeneity across investigation populations [8]. Based on cross-sectional populations, *Toxoplasma gondii *seropositivity has also been studied in terms of the association between serious mental illness and its severity, though causality has not been established [9]. Biological plausibility has been probed in experimental investigations, including evidence that T*oxoplasma gondii* infection could affect dopamine metabolism [10].

Narrative and immunological reviews bringing together these epidemiological and mechanistic findings have urged that latent toxoplasmosis should be understood as a vulnerability factor for other genetic and environmental risks that influence psychotic illnesses, rather than a direct causative factor for psychotic disorders [11]. More recently, longitudinal cohorts have provided evidence that *Toxoplasma gondii* exposure may precede disease onset in schizophrenia, supporting a contributory as opposed to an aetiological role [12,13]. Other possible mechanisms include immune-mediated neuroinflammation and general neurotransmitter dysregulation, although these hypotheses have not been proven for humans [14,15]. Published case reports of *Toxoplasma gondii*-mediated psychosis in immunocompetent adults are infrequent and usually present atypical clinical characteristics or imaging features that were not observed in this study [5,6]. In established schizophrenia, latent *Toxoplasma gondii *seropositivity has also been noted in association with cognitive dysfunction, even if no active infection is present [16]. The cumulative evidence favours latent toxoplasmosis as a potential risk modulator rather than a sufficient or single cause, and the meta-analytic results among psychiatric samples all agree with caution in interpreting the findings at the bedside [17].

In this case, co-existing risk factors like trauma, stimulant and alcohol use, and psychosocial stressors are probably more directly contributing to the patient’s clinical presentation. Existing clinical guidelines do not recommend the use of antiparasitic drugs for latent *Toxoplasma gondii *infection in immunocompetent individuals presenting with psychosis, and the best management is standardised antipsychotic and psychosocial interventions [18].

This case highlights the need for caution in interpreting incidental serological findings in complex psychiatric presentations and to avoid misattributing a causal relationship.

## Conclusions

Incidental serological findings, such as latent *Toxoplasma gondii* infection, should be interpreted cautiously in acute psychosis and do not, in immunocompetent patients, implicate an organic aetiology. Associations between latent toxoplasmosis and psychotic disorders reflect population-level risk and should not be taken to imply causation at the individual patient level. In contrast, substance misuse and psychosocial stressors can significantly exacerbate psychotic relapse and should be actively assessed and addressed in both formulation and management. Long-acting injectable antipsychotics may be beneficial in patients with recurrent relapse related to poor adherence. There is no evidence to support antiparasitic treatment for latent *Toxoplasma gondii* infection in immunocompetent patients presenting with psychosis.
